# Editorial: Co-morbid substance use and family violence

**DOI:** 10.3389/fpsyt.2025.1689029

**Published:** 2025-09-16

**Authors:** Ashlee Curtis, Richelle Mayshak, Cory A. Crane

**Affiliations:** ^1^ School of Psychology, Deakin University, Geelong, VIC, Australia; ^2^ Department of Psychiatry, Addiction Psychiatry, University of Rochester Medical Center, Rochester, NY, United States

**Keywords:** substance use, family violence, intimate partner violence, intervention, screening

The relationship between substance use and aggressive or violent behaviour has been consistently established (1). Despite this, there remain high levels of comorbidity for these behaviours. While research often considers substance use as a proximal factor in violent behaviour, studies often lack ecological validity such that the role of substance use in family violence, in particular, remains unclear (2, 3). As a consequence, efforts to establish consistent identification, appropriate screening tools, and treatment and intervention options within this population continue to lag.

Substance use can both trigger and intensify instances of family violence (4, 5) while experiences of family violence can contribute to the development and perpetuation of substance use disorders (6). This bi-directional relationship is influenced by a range of factors that are explored in this Research Topic including experiences of trauma, systemic barriers impacting identification of the role of substance use in family violence, and effective intervention.

## Mapping the landscape: scope and prevalence

Beeler et al. conducted a systematic mapping review that highlighted the diversity of violence experiences among individuals accessing substance use treatment. The review identified a lack of focus on specific subsets of the population, including, but not limited to male samples, parents, individuals within correctional facilities, and gender diverse couples. This indicates a need for more inclusive and representative research that attends to intersectional risk factors and system-level inequities. Importantly, Beeler et al. identified the necessity of treatment for substance use, histories of violence, and trauma to be integrated, and that such treatments must account for client and family level factors that may create barriers to accessible, responsive, and effective systems of care.

Addressing one of the under-researched subgroups identified by Beeler et al., Yoon et al. used nationally representative U.S. child welfare data to estimate caregiver substance dependence rates. They identified that caregiver substance use was strongly linked with domestic violence and caregiver depression, reinforcing the intergenerational and compounding impacts of comorbidity. Their findings also offer support to the recommendations of Beeler et al. that there is a need for integrated treatment options that address common co-morbidities, such as the intersection between child maltreatment and caregiver substance use.

## Mechanisms and screening challenges

Jarnecke and Saraiya reviewed IPV screening and referral practices in clinical settings. They found that while validated IPV screening tools and referral programmes exist, their implementation is inconsistent, particularly in substance use disorder treatment clinics. The authors attributed this inconsistency to a lack of available services when screening identifies a concern, a lack of practitioner education and awareness of the comorbidity between substance use and IPV in substance use treatment settings, short treatment durations, and the exclusion of persons who engage in IPV from substance use treatment. Echoing Beeler et al.‘s call for integrated interventions, this review highlights the need for more comprehensive, trauma-informed models of care that are embedded in routine practice, including consistent screening practices for both the use and experience of IPV in substance use treatment settings. The implementation of such models requires appropriate education and training for practitioners to ensure effective uptake.

## Intervention innovation and implications

Lila et al. offered promising evidence for motivational strategies in court mandated intervention programmes. The authors noted that among men who were court mandated to attend treatment for IPV, those with alcohol and other drug use problems were more likely to drop out of treatment and therefore received a lower treatment dose, and had higher levels of recidivism than those without alcohol and other drug use problems. However, when the standard intervention was combined with an individualised motivational plan, the differences in dropout rates, dosage, and recidivism were mitigated, whereby there was no difference observed between individuals with alcohol and drug use problems and those without. This supports prior evidence that motivational interviewing can enhance engagement for men in IPV intervention programmes (7). Importantly, their work drew attention to the often-overlooked treatment needs of perpetrators with substance use comorbidity, who are frequently excluded from intervention programmes (8). Lila et al.‘s findings suggest that motivational strategies may enhance treatment outcomes by increasing participant engagement.

## Conclusions and future directions

This Research Topic collectively reinforces the need for dual-focused, trauma-informed, and inclusive responses to the co-occurrence of substance use and family violence. Findings suggest that services remain siloed despite broad recognition of the association between substance use and IPV in the literature, screening is inconsistent, and workforce capacity remains underdeveloped. However, the articles in this Research Topic provide some ways forward for the field, identifying promising options for screening and intervention.

However, there remain key gaps that future research should prioritise to ensure that the co-occurrence of substance use and IPV can be effectively addressed. These include: (1) longitudinal designs to clarify causal pathways and intervention outcomes; (2) the development of culturally responsive screening and referral models; (3) intervention trials that are inclusive of subgroups under-represented in the literature; (4) effective implementation strategies to train providers and deploy efficacious resources; and (5) cross-sector collaboration to align funding and governance for family violence and substance use services.

## References

[1] Cafferky BM Mendez M Anderson JR Stith SM. Substance use and intimate partner violence: A meta-analytic review. Psychol Violence. (2018) 8:110–31. doi: 10.1037/vio0000074

[2] Capezza NM Schumacher EC Brady BC. Trends in intimate partner violence services provided by substance abuse treatment facilities: findings from a national sample. J Family Violence. (2015) 30:85–91. doi: 10.1007/s10896-014-9649-7

[3] Romo-Avilés N Tarriño-Concejero L Pavón-Benítez L Marín-Torres J. Addressing gender-based violence in drug addiction treatment: a systematic mapping review. Int J Ment Health Addict. (2024) 22:3656–82. doi: 10.1007/s11469-023-01072-4

[4] Curtis A Vandenberg B Mayshak R Coomber K Hyder S Walker A. Alcohol use in family, domestic and other violence: Findings from a cross-sectional survey of the Australian population. Drug Alcohol Rev. (2019) 38:349–58. doi: 10.1111/dar.12925, PMID: 30942525

[5] Schick MR Clayton A Maxwell CD Sullivan TP. Patterns of substance-involved intimate partner violence perpetration: Findings from a daily diary study. Addictive Behav. (2025) 166:108305. doi: 10.1016/j.addbeh.2025.108305, PMID: 40049052 PMC11968212

[6] Ogden SN Dichter ME Bazzi AR. Intimate partner violence as a predictor of substance use outcomes among women: A systematic review. Addictive Behav. (2022) 127:107214. doi: 10.1016/j.addbeh.2021.107214, PMID: 34933089 PMC10007694

[7] Musser PH Semiatin JN Taft CT Murphy CM. Motivational interviewing as a pregroup intervention for partner-violent men. Violence Vict. (2008) 23:539–57. doi: 10.1891/0886-6708.23.5.539, PMID: 18958984

[8] Radatz DL Wright EM. Integrating the principles of effective intervention into batterer intervention programming: the case for moving toward more evidence-based programming. Trauma, Violence, & Abuse. (2015) 17(1):72–87. doi: 10.1177/1524838014566695, PMID: 25573844

